# Etiopathogenesis of cataract: Journal review

**Published:** 2009

**Authors:** Rajesh Sinha, Chandrashekhar Kumar, Jeewan S Titiyal

**Affiliations:** 492, R. P. Centre for Ophthalmic Sciences, All India Institute of Medical Sciences, Ansari Nagar, New Delhi, India

Cataract is the leading cause of blindness all over the world. An exact etiology and mechanism of cataract formation may be difficult to ascertain in most of the cases. However, many mechanisms of cataractogenesis have been proposed and reported in the literature. We have made an attempt to review some of the published articles related to the etiopathogenesis of cataract.

## Congenital cataract

**El Fkih *et al.*** (*Tunis Med. 2007;85(12):1025-9*) studied the etiologies of congenital cataract in 85 children in a cross sectional study. An etiology of congenital cataract was found in 62.5% of cases. A hereditary factor was the most common cause; it constituted 42.3% of etiologies. Among these cases, 77.7% were autosomal recessive. Sixteen percent of congenital cataracts were associated with systemic diseases or dysmorphology syndromes. Metabolic diseases and intrauterine infections were found in 7% and 4.7% of cases respectively.

**Haargaard *et al.*** (*Ophthalmology 2004;111(12):2292-8*) studied the distribution of congenital/ infantile cataract in children (0 to 17 years old) who were born between 1959 and 2001 and registered in Danish National Register of Patients. Data of 1027 children with congenital/infantile cataract (529 boys and 498 girls) of whom 64% were bilateral were evaluated. Males predominated with bilateral cataract, whereas females predominated with unilateral cases. Isolated cataract was the most frequent clinical presentation (71% of all cases), followed by an even proportion of cataract associated with additional ocular dysmorphology and cataract associated with systemic anomalies. Almost two thirds of all cases had an unknown etiology (idiopathic). Idiopathic cases showed a higher proportion of unilateral cataract and of additional ocular dysmorphology compared with cases of known etiology. The etiology was unknown in 87% of unilateral cases and in 50% of bilateral cases. The distribution by presumed etiology was stable during the study period, except for cataract caused by maternal infections, which decreased mainly due to the elimination of congenital rubella.

**Haargaard *et al.*** (*Invest Ophthalmol Vis Sci. 2005;46(9):3067-73*) also investigated maternal, demographic, and pre- and perinatal risk factors for idiopathic congenital/ infantile (ICI) cataract. Based on national registries, a cohort of all children born in Denmark and aged 0 to 17 years during 1977 to 2001 was established, and congenital/infantile cataract cases were identified. Bilateral isolated cataract cases were male dominated (62%; 95% confidence interval [CI], 56%-69%) but not unilateral isolated cases (40%; 95% CI, 34%-47%). Older age (≥ 40 years) of mothers at delivery increased the risk of ICI cataract. Low birth weight (< 2000 g) was associated with a 10.6-fold (95% CI, 6.99-16.10) increased risk of bilateral, but not unilateral, ICI cataract. No significant associations were found with birth order, month/ place of birth, or cigarette smoking during pregnancy. Low-birth-weight children (< 2000 g) had a significantly increased risk of bilateral ICI, whereas no strong risk factors were found for unilateral cataract.

**Rahi *et al.*** (*Invest Ophthalmol Vis Sci. 2000;41(8):2108-14*) studied the association/ risk factors of congenital and infantile cataract in United Kingdom. Of 243 children with newly diagnosed congenital or infantile cataract, 160 (66%) had bilateral disease. Isolated cataract was more common in bilateral than unilateral cases (61% versus 47%, p = 0.05) as was cataract associated with a systemic disorder (25% versus 6%, *P* < 0.001). Conversely, cataract with associated ocular anomalies was more common in unilateral than bilateral cases (47% versus 14%, *P* < 0.001). No underlying or associated risk factors for cataract could be identified in 92% of unilateral and 38% of bilateral cases, although putative prenatal and perinatal risk factors were reported in a proportion of these idiopathic cases. Hereditary disease was associated with 56% of bilateral but only 6% of unilateral cases. Prenatal infections and other systemic factors were reported in only 6% of bilateral and 2% of unilateral cases.

**SanGiovanni *et al.*** (*Arch Ophthalmol. 2002;120(11):1559-65*) estimated the prevalence of 4 categories of infantile cataract in subjects surviving the neonatal period in a US cohort, and investigated risk factors for isolated infantile cataract. Infantile cataract occurred in 13.6 per 10000 infants. Isolated infantile cataract occurred 3.8 times as often among infants born at weights at or below 2500 g than among those born at or above 2500 g (95% CI, 1.5-8.6; P<.001); after controlling for a set of covariates, similar results were observed for bilateral isolated cataract (odds ratio = 4.4; 95% CI, 1.2-13.9). They concluded that infants born at weights at or below 2500 g were found to have 3- to 4-fold increased odds of developing infantile cataract.

**Adhikari *et al.*** (*JNMA J Nepal Med Assoc. 2007;46(167):94-8*) performed a hospital based cross sectional study in Nepal regarding clinical profile and etiology of cataract in pediatric age group with an emphasis on preventable factors. Out of 172 children, 34 (88%) had non-traumatic and 65 (12%) children had traumatic cataract. The mean age was 5.63 ± 3.59 years and 7.39 ± 3.94 years in non-traumatic group and traumatic group respectively. Among non-traumatic cataract, 42 (37.50%) had hereditary, 9 (8.03%) had systemic syndromes, 5 (4.46%) had cataract due to maternal infection, and 3 (2.67%) had metabolic disorder; 11 (9.82%) cataract were associated with other ocular dysmorphology, and the cause was not ascertained in 31 cases. Eleven (9.82%) cases had complicated cataract. Twenty seven (24.1%) children with bilateral cataract had nystagmus at the time of presentation. In traumatic group, play related injuries were more common than household injuries.

**Johar *et al.*** (*Indian J Med Sci. 2004;58(3):115-21*) surveyed the causes of childhood cataracts and identified the preventable factors in four western states of India. Out of 172 children, 88.4% had non-traumatic cataract and 11.6% had traumatic cataracts. Among non-traumatic cataracts, 7.2% were hereditary, 4.6% were due to congenital rubella syndrome, 15.1% were secondary and 73.0% were undetermined. In the group of undetermined cases, 67% of the mothers had history of illness during pregnancy, and 22% had taken medications during pregnancy. This study showed that nearly 12% of non-traumatic cataract was due to potentially preventable causes.

**Eckstein *et al.*** (*Br J Ophthalmol.1996;80(7):628-32*) evaluated 514 consecutive children with cataract and studied factors that might be potentially preventable. Of the 366 children with non-traumatic cataract 25% were hereditary, 15% were due to congenital rubella syndrome (CRS), and 51% were undetermined. In children under 1 year of age, 25% were due to rubella, and cataract of nuclear morphology had a 75% positive predictive value for CRS. Mothers of children in the undetermined group were more likely to have taken abortifacients than a group of age matched controls (p = 0.1) but use of other medications in pregnancy was similar in both groups. Of the 148 (29%) children with traumatic cataracts three quarters were over the age of 6 years. Stick injuries were responsible for 28%, thorn injuries for 21%, and firecrackers for 5%. Nearly half of non-traumatic cataract in south India was due to potentially preventable causes.

## Metabolic diseases and cataract

**Wijburg *et al.*** (*Ned Tijdschr Geneeskd. 2008;152(11):632-6*) described three young patients who presented with bilateral cataracts, the cause proved to be an inherited metabolic disease. The first patient was a newborn aged 7 weeks, in whom galactokinase deficiency was diagnosed. The second patient was a boy aged 8 years with cerebrotendinous xanthomatosis. The third patient was a girl who was diagnosed with cataracts at the age of 3 months. At the age of 4 years the diagnosis ‘rhizomelic chondrodysplasia punctata’ was established. They concluded that screening for metabolic disorders in all children with bilateral cataracts is essential. The diagnosis also allows for family studies and genetic counseling to take place.

**Olsen** *et al.* (*Ophthalmic Paediatr Genet.1993;14(2):87-9*) described three siblings with Morquio syndrome (mucopolysaccharidosis IV A) with cataract. In addition to the characteristic dwarfism with skeletal deformities, odontoid anomalies, hearing loss and corneal clouding, the authors found almost identical lens opacities in all three patients.

**Sulochana** *et al.* (*Br J Ophthalmol.1997;81(4):319-23*) in their study reported congenital or infantile cataract with deranged metabolism of proteoglycans (acid mucopolysaccharide-xylose-protein complex). They observed that an increase in beta glucuronidase activity might have caused extensive fragmentation of glycosaminoglycans with resultant accumulation in the blood and lens.

## Steroid induced cataract

### Oral steroid

**Wang *et al.*** (*Ophthalmology 2009 Feb 23*) studied longitudinal associations between inhaled and oral corticosteroid use and 10-year incidence of cataract. Current users had a greater risk of developing posterior sub-capsular (PSC) cataract and nuclear cataract but not cortical cataract. Interaction between inhaled and oral corticosteroid use was significant for PSC (*P* = 0.01) and nuclear (*P* = 0.02) cataract incidence. In subgroup analyses, only individuals who used both inhaled and oral steroids were at increased risk of PSC cataract.

### Posterior subtenon injection

**Yoshimura *et al.*** (*Nippon Ganka Gakkai Zasshi. 2008;112(9):786-9*) conducted a retrospective, case-control study in 44 phakic eyes of 38 patients who underwent administration of triamcinolone acetonide. Eight eyes (18%) had progression of PSC cataract during 12 months after triamcinolone injection. Five eyes (11%) required cataract surgery. The mean time to the cataract progression was 8.8 ± 3.7 months.

### Inhalational steroids

**Behbehani *et al.*** (*J Pediatr Ophthalmol Strabismus. 2005;42(1):23-7*) studied the incidence of PSC cataract and ocular hypertension (OH) in a cohort of children ≤ 12 years on inhaled steroid therapy. Ninety patients underwent eye examination 2 years later; none was found to develop cataract or increased intraocular pressure (IOP).

### Pulse steroid

**Nerome *et al.*** (*Pediatr Int. 2008;50(4):541-5*) studied the relationship between the formation of cataract and steroid therapy in patients with rheumatic disease. The initiation of steroid therapy in children under 12 years of age (*P* = 0.041) and intravenous methylprednisone pulse therapy (IVMP) (*P* = 0.046) were found significant risk factors for inducing cataracts. In contrast, the cumulative corticosteroid dose, sex, and daily corticosteroid dose were not associated with cataract formation.

### Intravitreal steroid injection

**Chu *et al.*** (*Eye. 2008;22(7):895-9*) investigated the progression of cataract after a high dose (25 mg) intravitreal triamcinolone acetonide injection in 38 patients (76 eyes) with macular edema secondary to diabetes and retinal vein occlusion and reported progression of cortical and posterior subcapsular opacity in these patients.

**Islam *et al.*** (*Eye 2007;21(3):321-3*) determined the incidence of cataract following intravitreal triamcinolone (IVTA) for diabetic macular edema in 27 eyes of 27 patients. Twenty two (81%) eyes developed cataract during the follow-up period, of which 20 (74%) were posterior subcapsular in nature. None of the 20 uninjected fellow eyes developed posterior subcapsular cataract. Mean time to cataract formation was 16.2 months.

**Thompson** (*Am J Ophthalmol. 2006;141(4):629-37*) evaluated complications and results of IVTA for treatment of macular edema. They concluded that IVTA improves visual acuity in most eyes but eyes must be monitored carefully for increase in IOP. Posterior subcapsular cataract becomes visually significant in almost half of eyes by one year after injection.

**Cekiç** *et al.* (*Am J Ophthalmol. 2005;139(6):993-8*) performed a similar study and concluded that single IVTA injection induces PSC cataract development, whereas multiple injections result in all-layer cataract progression.

### Corticosteroid Enema

**Patteau** *et al.* (*J Fr Ophtalmol. 2003;26(8):834-6*) reported a 65-year-old patient suffering from a long-term ulcerative colitis treated with corticosteroid enema and presenting with a bilateral PSC cataract.

## Radiation

**Tichelli *et al.*** (*Ann Intern Med. 1993 15;119(12):1175-80*) evaluated the incidence, time course, and factors associated with cataract formation in bone marrow transplant (BMT) recipients. Three regimens for bone marrow transplant were used: 74 patients received single-dose, total-body irradiation (TBI), 90 patients received fractionated TBI, and 33 received chemotherapy alone. They observed that the incidence of cataract is higher and lens opacification occurs earlier after single-dose TBI than after fractionated TBI (*P* < 0.01). The probability of requiring cataract surgery after 6 years was 85% for the patients treated with single-dose TBI and 20% for those prepared with fractionated irradiation.

**Benyunes *et al.*** (*Int J Radiat Oncol Biol Phys. 1995;32(3):661-70*) determined the risk of, and risk factors for, developing cataracts after bone marrow transplantation in 492 adults. One hundred and fifty-nine patients (32%) developed cataract between 0.5 and 11 (median, 2.3) years after transplantation. The probability of cataracts at 11 years after transplantation was 85%, 50%, 34%, and 19% for patients receiving 10 grey (Gy) of single-dose TBI, > 12 Gy fractionated TBI, 12 Gy fractionated TBI, and no TBI, respectively (*P* < 0.0001). Among those developing cataracts, the severity was greater in patients after single-dose TBI (59% probability of surgical extraction) than after > 12 Gy fractionated TBI, 12 Gy fractionated TBI, or no TBI (33%, 22% and 23%, respectively). Patients given corticosteroids after transplant had a higher probability of cataract (45%) than those without steroids (38%) (*P* < 0.0001). The yearly hazard of developing cataract in recipients of single-dose TBI was highest during the third year after transplantation, while in recipients of fractionated TBI, the hazard was distributed among years one through seven.

## Pseudocataract and ivta

**Jain *et al.*** (*Ann Ophthalmol (Skokie) 2006;38(1):67-8*) described formation of pseudo-cataract by the adherence of triamcinolone particles to the posterior surface of the lens following a 2-mg intravitreal injection for treatment of macular edema associated with non-ischemic central retinal vein occlusion.

## Post surgical

### Post trabeculectomy

**Adelman *et al.*** (*Ophthalmology 2003;110(3):625-9*) evaluated the risk of cataract formation in young patients after initial trabeculectomy. The rate of cataract extraction after initial trabeculectomy was 24% (n = 8). The average time from trabeculectomy to cataract extraction was 26 months (range, 5-58 months).

**Advanced Glaucoma Intervention Study (AGIS) Investigators** (*Arch Ophthalmol. 2001;119(12):1771-9*) compared the risk of cataract formation in eyes with and without prior trabeculectomy and assessed other risk factors for cataract. They concluded that in eyes of AGIS patients, after adjustment for age and diabetes, trabeculectomy increased the risk of cataract formation by 78%.

### Post vitreoretinal surgery

**Smiddy *et al.*** (*Retina 2004;24(4):574-81*) determined the frequency of visually significant cataracts after vitrectomy for complications of diabetic retinopathy in 40 patients and 56 concurrent controls with the diagnosis of macular hole or epiretinal membrane. They concluded that the rate of cataract extraction after vitrectomy in patients with diabetes is lower than in patients without diabetes undergoing vitrectomy. This inference should be considered when attributing subnormal vision to cataract in a patient who had had a diabetic vitrectomy.

**Thompson *et al.*** (*Am J Ophthalmol. 2004;137(2):250-7*) evaluated the rate of increase in nuclear sclerosis and PSC cataracts in eyes as a function of patient age and use of intravitreal gas at the time of vitrectomy in 301 consecutive eyes. He concluded that intravitreal gas bubbles are associated with a nuclear sclerosis increase of approximately 60% compared with eyes without use of a gas bubble.

**Thompson *et al.*** (*Am J Ophthalmol.1995;119(1):48-54*) also studied the progression of cataracts and visual acuity up to 36 months after vitrectomy and instillation of transforming growth factor beta-2 for treatment of full-thickness macular holes. Nuclear sclerotic cataracts progressed substantially after macular hole surgery with a long-acting intraocular gas tamponade. The visual acuity often decreased 12 or more months after vitrectomy because of cataract progression, but the visual results of vitrectomy and transforming growth factor beta-2 for macular holes were excellent when the cataracts were removed.

## Effect of medication

**Klein *et al.*** (*Ophthalmology 2001;108(9):1670-4*) evaluated incident cataract after a 5-year interval with respect to various medication use. Significantly lower incidences of nuclear cataracts occurred in those who took thiazide diuretics and aspirin at the baseline examination. There were significantly more incident cortical cataracts in those taking oral steroids, amitriptyline, oral hypoglycemic agents, and insulin and more incident PSC cataracts in those taking potassium-sparing diuretics and oral hypoglycemic agents.

### Role of aspirin

**Christen *et al.*** (*Arch Ophthalmol. 2001;119(3):405-12*) in a randomized trial reported that there was no major beneficial effect of 5 years of low-dose aspirin treatment on total cataract.

### Role of ß- Carotene

**Christen *et al.*** (*Arch Ophthalmol. 2003;121(3):372-8*) examined the development of age-related cataract in a trial of beta carotene supplementation in men. Data suggested no overall benefit or harm of 12 years of beta carotene supplementation in terms of cataractogenesis or cataract extraction. However, among current smokers at baseline, beta carotene appeared to attenuate their excess risk of cataract by about one fourth.

**Christen *et al.*** (*Ophthalmic Epidemiol. 2004;11(5):401-12*) in another study examined the development of age-related cataract in a trial of beta-carotene supplementation in women for two years and reported no significant beneficial or harmful effect.

## Secondary to other ocular pathology

### Uveitis and Cataract

**Malinowski *et al.*** (*Ophthalmology1993;100(6):818-24*) described long-term visual outcome and complications associated with pars planitis. They found visually significant cataracts in 14.8% cases of pars planitis.

**Donaldson *et al.*** (*Am J Ophthalmol. 2007;144(6):812-817*) in their study reported an incidence of 30.4% of complicated cataract in a cohort of cases of pars planitis.

### Glaucoma

Chandrasekaran *et al.* (*Ophthalmology 2006;113(3):417-24*) studied incident relationships between elevated IOP, open-angle glaucoma (OAG), and use of glaucoma medications with 5-year incidence of cataract. The 5-year incidence of nuclear cataract was 23.4% (592/2532), or 23.1% (574/2486) after excluding subjects using glaucoma medication. A marginally significant association was found for elevated IOP and incident nuclear cataract in subjects not using glaucoma medications, after multivariate adjustment. Use of glaucoma medications was associated with non-significantly increased adjusted odds for incident nuclear cataract (OR, 1.90 [95% CI, 0.92-3.92]). No associations, however, were found with incident cortical cataract or PSC. They concluded that elevated IOP may increase the risk of nuclear cataract, but not that of other types. The use of glaucoma medications could magnify this risk.

### Retinal lesions

**Tan *et al.*** (*Ophthalmology 2008;115(10):1693-8*) determined whether local nutritional or ischemic factors e.g. narrowed retinal vessel caliber predicted the long-term incidence of age-related cataract. They concluded that retinal vessel narrowing predicted greater risk of long-term incidence of PSC cataract and cataract surgery, and were indirectly linked to a lower incidence of nuclear cataract. Retinal vessel narrowing could be a marker of age-related factors associated with risk of PSC and nuclear cataract.

### Refractive error

**Lim *et al.*** (*Invest Ophthalmol Vis Sci. 1999;40(12):3021-6*) assessed the relationship between myopia and age-related cataract in a defined older population. Eyes with onset of myopia before 20 years had the greatest risk of PSC cataract (OR 3.9; CI 2.0-7.9). Posterior subcapsular cataract was inversely associated with hyperopia (OR 0.6; CI 0.4-0.9). High myopia was associated with PSC, cortical, and late nuclear cataract. They concluded that early-onset myopia (before age 20 years) may be a strong and independent risk factor for PSC cataract.

**Younan *et al.*** (*Invest Ophthalmol Vis Sci. 2002;43(12):3625-32*) in a similar study reported a statistically significant association of high myopia with incident nuclear cataract.

### Iris colour

**Younan *et al.*** (*Am J Ophthalmol. 2002;134(2):273-4*) assessed whether an association exists between iris color and the incidence of cataract and cataract surgery. Participants with dark brown iris color had an increased incidence of nuclear cataract, and cataract surgery, compared with participants with blue iris color.

**Cumming *et al.*** (*Am J Ophthalmol. 2000;130(2):237-8*) in a similar study reported that eyes with dark brown irises were more likely to have nuclear or PSC cataract than eyes with lighter-colored irises and should be encouraged to protect their eyes from direct exposure to sunlight.

## Systemic condition

### Dermatitis

**Taniguchi *et al.*** (*J Dermatol. 1999;26(10):658-65*) evaluated ocular complications in patients being treated for severe atopic dermatitis. Cataract was found in 20 cases (25.3%), and retinal detachment in 9 (11.4%) cases. The development of cataract or retinal detachment had no relationship to serum IgE levels, personal history of respiratory atopy, the duration of topical corticosteroid use on the face, or treatment with systemic corticosteroid.

**Katsushima *et al.*** (*Nippon Ganka Gakkai Zasshi. 1994;98(5):495-500*) performed a similar study with 75 patients. Cataract was found in 13 (9 bilateral and 4 unilateral) patients, and retinal detachment in 6 patients (3 bilateral & 3 unilateral).

### Diabetes and Hypertension

**Younan *et al.*** (*Ophthalmic Epidemiol. 2003;10(4):227-40*) assessed whether an association exists between cardiovascular disease, vascular risk factors and incident cataract and cataract surgery. They found that hypertensive patients using medication and aged less than 65 years at baseline had a higher incidence of PSC cataract (OR 3.4, 95% CI 1.3-8.4) than normotensive subjects. A history of angina was associated with higher cataract surgery incidence (OR 2.1, 95% CI 1.3-3.5).

**Tan *et al.*** (*Ophthalmic Epidemiol. 2008;15(5):317-27*) assessed associations between diabetes and selected cardiovascular risk factors and long-term incident cataract and cataract surgery. Higher body mass index (BMI) was positively associated with PSC cataract (RR per SD, 1.20; CI, 1.03-1.41). Persons using anti-hypertensive medication had a higher incidence of cataract surgery (RR, 1.61; CI, 1.18- 2.20). Metabolic syndrome was associated with an increased risk of all 3 cataract subtypes. A cluster of metabolic abnormalities attributable to insulin resistance appeared more likely to contribute to cataract formation than any individual cardiovascular risk factor alone.

**Delcourt *et al.*** (*Am J Epidemiol. 2000 1;151(5):497-504*) observed an increased risk of cataract in females, brown iris, smokers, known diabetic for more than 10 years, those using oral corticosteroids for at least 5 years, asthma or chronic bronchitis, cancer and cardiovascular disease. Decreased risk of cataract was found with higher education, hypertension, and high plasma retinol levels.

### Smoking and Cataract

**Tan *et al.*** (*Ophthalmic Epidemiol. 2008;15(3):155-61*) assessed the association between smoking and the long-term incidence of cataract and cataract surgery. The effect of smoking was strongest in ever smokers reporting 36 + pack-years of smoking compared to never smokers (RR 1.46; CI, 1.02-2.08). Current smokers also developed nuclear cataract slightly earlier than non-smokers (mean age 65.2 versus 67.5 years, *P* = 0.049). No statistically significant associations were found between smoking status and the incidence of cortical or PSC cataract, or cataract surgery.

**Christen *et al.*** (*JAMA 1992;268(8):989-93*) examined the association between cigarette smoking and the incidence of cataract. After controlling for other potential risk factors, current smokers of 20 or more cigarettes per day had statistically significant increases in nuclear sclerosis and PSC cataract. Past smokers had an elevated risk of PSC but not nuclear cataract. For current smokers of fewer than 20 cigarettes per day, no increased risks were observed.

**Christen *et al.*** (*JAMA2000;284(6):713-6*) in another study examined the association between smoking cessation and incidence of age-related cataract. They reported that while some smoking-related damage to the lens may be reversible, smoking cessation reduces the risk of cataract primarily by limiting total dose-related damage to the lens.

### Alcohol and Cataract

**Lindblad *et al.*** (*Ophthalmology 2007;114(4):680-5*) in their study noted that daily intake of ≥1 alcoholic drinks was associated with a modest increase of risk for cataract extraction. The risk increased with increasing alcohol consumption.

**Chasan-Taber *et al.*** (*Ann Epidemiol. 2000;10(6):347-53*) in a similar study noted that there was no substantial overall increased risk of senile cataract due to alcohol intake.

### Body Mass Index (BMI) and Cataract

**Glynn *et al.*** (*Arch Ophthalmol. 1995;113(9):1131-7*) in their study reported that higher BMI was especially strongly related to risk of PSC and nuclear cataracts. They noted that the effect of BMI on cataractogenesis was apparently independent of other risk factors, including age, smoking, and diagnosed diabetes.

## Sunlight exposure

**Neale *et al.*** (*Epidemiology 2003;14(6):707-12*) in a study reported a strong positive association of occupational sun exposure between the ages of 20 and 29 years with nuclear cataract (OR 5.9; 95% CI, 2.1- 17.1). Exposure later in life resulted in weaker associations.

**Delcourt *et al.*** (*Arch Ophthalmol. 2000;118(3):385-92*) studied the relationship of ambient solar radiation and professional and leisure exposures to light with the different types of cataracts. They reported that participants who had higher ambient solar radiation had a 2.5-fold, 4.0-fold, and 2.9-fold increased risk of cortical and mixed cataract and cataract surgery, respectively. Solar ambient radiation was not significantly associated with PSC and nuclear cataracts. By contrast, PSC cataracts were significantly associated with professional exposure to sunlight and frequent use of sunglasses. Mixed cataract was also associated with professional exposure to artificial light.

**Pastor-Valero *et al.*** (*BMC Ophthalmol. 2007 26;7:18*) also investigated the relationship between sunlight exposure and risk of cataract. No association was found between years of outdoor exposure and risk of cataract. However, exploratory analyses suggested a positive association between years of outdoor exposure at younger ages and risk of nuclear cataract later in life.

## Oxidative stress and cataract

**Truscott** (*Int J Biochem Cell Biol. 2003;35(11):1500-4*) studied the mechanisms responsible for age related cataract. They observed that the centre of the lens contains proteins that were synthesized prior to birth and while these crystallins are remarkably stable, it appears that an antioxidant environment may be necessary in order for them to remain soluble and for lens transparency. Once an internal barrier to the movement of small molecules, such as antioxidants, develops in the normal lens at middle age, the long-lived proteins in the lens centre become susceptible both to covalent attachment of reactive molecules, such as ultraviolet (UV) filters, and to oxidation. These processes of protein modification may, over time, lead inevitably to lens opacification and cataract.

**Ottonello *et al.*** (*Ophthalmologica2000;214(1):78-85*) also reported an association of oxidative stress with cataract formation, in their study.

**Dillon** (*Doc Ophthalmol. 1994-1995;88(3-4):339-44*) observed that the functions of the human lens is to filter light between 300-400 nm from reaching the retina. The lens is therefore continuously under photo-oxidative stress. Photophysical studies have demonstrated that in the long term this can lead to yellowing of lens proteins. The yellowing of lens proteins leads to a drastic increase in the number of photons absorbed by the lens. This, along with the age-related losses of antioxidants increases the photo-oxidative stress on the lens, thereby increasing the risk of cataractogenesis.

## Socioeconomic status and cataract

**Kuper *et al.*** (*PLoS Med. 2008;5(12):e244*) examined the association between visual impairment from cataract and poverty in adults in Kenya, Bangladesh, and the Philippines. Data from this study showed that people with visual impairment due to cataract were poorer than those with normal sight in all three low-income countries studied.

